# Secular Trends of Incidence, Prevalence, and Healthcare Economic Burden in ANCA-Associated Vasculitis: An Analysis of the 2002–2018 South Korea National Health Insurance Database

**DOI:** 10.3389/fmed.2022.902423

**Published:** 2022-07-07

**Authors:** Sung Soo Ahn, Hyunsun Lim, Chan Hee Lee, Yong-Beom Park, Jin-Su Park, Sang-Won Lee

**Affiliations:** ^1^Department of Internal Medicine, Yongin Severance Hospital, Yonsei University College of Medicine, Yongin-si, South Korea; ^2^Research and Analysis Team, National Health Insurance Service Ilsan Hospital, Goyang-si, South Korea; ^3^Division of Rheumatology, Department of Internal Medicine, National Health Insurance Service Ilsan Hospital, Goyang-si, South Korea; ^4^Division of Rheumatology, Department of Internal Medicine, Yonsei University College of Medicine, Seoul, South Korea; ^5^Institute for Immunology and Immunological Diseases, Yonsei University College of Medicine, Seoul, South Korea

**Keywords:** antineutrophil cytoplasmic antibody-associated vasculitis, South Korea, incidence, prevalence, healthcare burden

## Abstract

**Objectives:**

The incidence and prevalence of AAV in Asia remain poorly understood, especially in a nationwide setting. This study investigated the incidence, prevalence, and healthcare burden of antineutrophil cytoplasmic antibody (ANCA)-associated vasculitis (AAV) in South Korea by analyzing a national database.

**Methods:**

This study included patients with AAV identified from the National Health Insurance Service Database of South Korea from 2002 to 2018. Patients were diagnosed with AAV in a general or tertiary hospital and were registered in the individual payment beneficiaries program or were prescribed glucocorticoids. A calendar-based meteorological definitions were adopted to assess the differences in the incidence of AAV according to season. The average healthcare expenditure and patient outcomes of mortality and end-stage renal disease (ESRD) in patients with AAV were compared to 1:10 age, sex and residential area matched controls.

**Results:**

A total of 2,113 patients [708, 638, and 767 with microscopic polyangiitis (MPA), granulomatosis with polyangiitis, and eosinophilic granulomatosis with polyangiitis, respectively] were identified. The annual incidence and prevalence of AAV increased continuously, and MPA being the most common disease subtype after 2015. The highest incidence and prevalence of AAV was 0.48/100,000 person-years (PY) and 2.40/100,000 PY in 2017 and 2018, respectively. There were no significant differences in monthly and seasonal incidence of AAV. The average expense of medical care, overall mortality, and ESRD rates of patients with AAV were higher in patients with AAV than in controls, especially in the case of MPA.

**Conclusion:**

An increasing trend of AAV diagnosis observed is consistent with the evidence that AAV is more common in recent years; however, a relatively lower incidence and prevalence was observed compared to that in Western countries. The higher medical cost and rates of mortality and ESRD in AAV emphasize the early recognition and implementation of optimal treatment for these patients.

## Introduction

Antineutrophil cytoplasmic antibody (ANCA)-associated vasculitis (AAV) is a potentially lethal systemic autoimmune disease affecting small-sized vasculatures, and comprises three subtypes: granulomatosis with polyangiitis (GPA), microscopic polyangiitis (MPA), and eosinophilic granulomatosis with polyangiitis (EGPA) (1). Detection of circulating ANCAs against myeloperoxidase (MPO) and proteinase 3 (PR3), although undetectable in ~20%−40% of patients (2), is a classic laboratory finding in AAV, and typical clinical and pathologic results are crucial for differentiating these disease subtypes (3). Judicious clinical suspicion is crucial in AAV diagnosis owing to the large heterogeneity of disease manifestations and absence of absolute pathognomonic features. Traditionally, AAV has been considered a rare disorder that usually occurs in the elderly population. However, an increasing number of evidence indicates that AAV is more common in recent years compared to the previous numerical estimates (4).

The incidence of AAV is largely variable according to the existing literature. A considerable disparity in its incidence has been reported based on the geographic region in which the research was conducted, with a higher incidence of AAV reported in Western countries and a much lower incidence in Asia (5). In addition, a predominance of GPA is observed in Northern Europe and Australia, whereas MPA has been reported as the most common disease subtype in Japan and Southern Europe (6). Meanwhile, US data demonstrated that MPA was the most frequent diagnosis among the incident cases of AAV (7). However, the prevalence of AAV appears to be similar to its incidence pattern in the corresponding region.

A previous study by Fujimoto et al., which investigated the incidence of AAV in regions of Asia, has shown that the average annual incidence of AAV was estimated to be comparable between Japan and the UK; however, MPA comprised most AAV cases in Japan, whereas GPA was predominantly found in the UK (8). Except for this study, no other studies have reported on the incidence and prevalence of AAV in Asia, especially in a nationwide population-based setting. Furthermore, as most epidemiologic studies of AAV were performed in Western countries and are scarce in other Asian countries, these data might insufficiently reflect the difference in global epidemiology; therefore, a better understanding is required. To address this issue, this study aimed to evaluate the incidence and prevalence in South Korea by analyzing a nationwide healthcare administrative database.

## Methods

### The National Health Information Database and Case Selection

We searched the National Health Information Database (NHID) from 2002 to 2018 for data acquisition. The NHID, which is managed by the South Korean government, contains information on the utilization of national hospital care of over 50 million individuals (more than 97% of South Korean residents) that is covered by the National Health Insurance Service (NHIS). As inclusion of the NHIS is mandated by the South Korean government, utilization of the NHID enables the identification of a large population of rare diseases in a nationwide healthcare setting suitable for epidemiological studies (9, 10). The dataset available in the NHID is as follows, which is also described elsewhere: (i) general information of patients such as age, gender, the healthcare provider, the date of visiting the hospital, and diagnosis requiring hospital care (either primary or secondary), (ii) utilization of healthcare services of procedures, operations, and injection, and (iii) the prescription of drugs for treatment (10). However, collecting detailed information of an individual is fundamentally prohibited, as it could result in de-identification of a patient.

In selecting patients with AAV, the following definitions were applied: (i) patients diagnosed with AAV in a general or tertiary hospital according to the ICD-10 codes for AAV (M31.7 for MPA, M31.3 for GPA and M30.1 for EGPA); (ii) those registered in the payment beneficiary program for rare and intractable diseases provided by the South Korean government with the corresponding code (V code) of AAV (V135 for MPA, V238 for GPA and V134 for EGPA); and (iii) those prescribed with glucocorticoids (prednisone, prednisolone, triamcinolone, methylprednisolone, dexamethasone, betamethasone, deflazacort, hydrocortisone and budesonide). Patients who satisfied definitions (i) + (ii) or (i) + (iii) were included for AAV case ascertainment.

In the present study, the date of AAV diagnosis (index date) was defined as the initial date of fulfilling the case ascertainment criteria. In addition, the incidence of AAV was calculated after applying a 2-year washout period (estimated from 2004), and prevalent cases were defined as those who fulfilled the criteria for case ascertainment and received outpatient or inpatient hospital care (estimated from 2002). Furthermore, as the ICD-10 code for MPA was included in the NHID in 2008, the incidence and prevalence of MPA were calculated after 2008. This study was approved by the Institutional Review Board of the NHIS Ilsan Hospital (Institutional Review Board No: 2022-01-003), and the requirement to obtain informed consent from the patients was waived due to the retrospective nature of the study.

### Baseline Variables, Medical Costs, and the Definition of Season

Baseline variables that were investigated at AAV diagnosis included sex, age, income and the type of insurance. The comorbidity of patients was evaluated using the Charlson comorbidity index (CCI) within 1 year of disease diagnosis (11). The average medical cost of patients with AAV was calculated as the total out-of-pocket expenses incurred in the hospital per patient after diagnosis.

In addition, calendar-based meteorological definitions of spring (1 March to 31 May), summer (1 June to 31 August), autumn (1 September to 30 November), and winter (1 December to 28 February) were adopted to assess the differences in the incidence of AAV according to season.

### Patient Outcomes and Selection of Controls

For patient outcomes, all-cause mortality and end-stage renal disease (ESRD) were investigated. Patient mortality was defined as the existence of a registered date of death after the onset of AAV, and those who were granted a medical expense reduction (V code) and treatment code (O code) for haemodialysis (V001 and O7020) and/or peritoneal dialysis (V003 and O7061) after AAV diagnosis were considered as developing ESRD (12). The follow-up duration was calculated as the time interval between disease diagnosis and the occurrence of death and ESRD in patients or the last follow-up date.

To compare the medical costs and patient outcomes between patients with AAV and the general population, 20,695 controls who received medical care at a general or tertiary hospital were randomly extracted by performing a 1:10 matching by age, sex and residential area.

### Statistical Analysis

Statistical analyses were conducted using the SAS V.9.4 Enterprise Guide (SAS Institute). Continuous and categorical variables were presented as mean (SD) and frequencies (percentages) and were compared using Student's *t*-test and Chi-squared or Fisher's exact test as indicated. The annual and prevalence rate/100,000 persons were calculated using the number of cases identified in the corresponding year and the population registered in the middle of each year. Differences in the trends of the incidence and prevalence of AAV were assessed using Poisson regression, and the influence of monthly and seasonal differences in disease incidence was calculated using a one-way analysis of variance test. A two-tailed *p*-value of <0.05 was considered significant in all statistical analyses.

## Results

### Comparison of Patient Characteristics Between Patients with AAV and Controls

Table 1 describes the baseline characteristics of the patients with AAV and the controls. The proportion of women and the mean age of patients with AAV were 53.9% and 58.1 years, respectively. AAV was most common in those aged 60–69 years. There was no difference in income status between patients with AAV and the controls. However, concerning insurance type, self-employment was more frequent in AAV patients, and the mean CCI was also higher in the AAV group.

**Table 1:** Baseline characteristics of patients with ANCA-associated vasculitis and controls.

	**Total**	**Controls**	**ANCA-associated vasculitis**	***p*-value**
	**(*n* = 22808)**	**(*n* = 20695)**	**(*n* = 2113)**	
**Sex**				
Male	10420 (45.7)	9445 (45.6)	975 (46.1)	0.658
Female	12388 (54.3)	11250 (54.4)	1138 (53.9)	
Age, years	57.9 ± 15.8	57.8 ± 15.8	58.1 ± 15.8	0.484
**Age distribution**				
10–19	373 (1.6)	339 (1.6)	34 (1.6)	0.997
20–29	1165 (5.1)	1059 (5.1)	106 (5.0)	
30–39	1701 (7.5)	1545 (7.5)	156 (7.4)	
40–49	2804 (12.3)	2547 (12.3)	257 (12.2)	
50–59	4813 (21.1)	4371 (21.1)	442 (20.9)	
60–69	5961 (26.1)	5413 (26.2)	548 (25.9)	
70–79	5064 (22.2)	4588 (22.2)	476 (22.5)	
80–89	895 (3.9)	804 (3.9)	91 (4.3)	
≥ 90	32 (0.1)	29 (0.1)	3 (0.1)	
**Income^†^**				
<3 rd quintile	4977 (22.3)	4511 (22.3)	466 (22.4)	0.624
3~7 th quintile	7198 (32.2)	6509 (32.1)	689 (33.1)	
>7 th quintile	10165 (45.5)	9236 (45.6)	929 (44.6)	
**Insurance type**				
Employee	9117 (40.0)	8430 (40.7)	687 (32.5)	<0.001
Self-employment	13005 (57.0)	11658 (56.3)	1347 (63.7)	
Medical-aid	686 (3.0)	607 (2.9)	79 (3.7)	
CCI	2.1 ± 1.3	2.0 ± 1.2	3.0 ± 1.6	<0.001

† *Data are available for 22,340 patients*.

*Data are expressed as mean (SD) or frequencies (percentages)*.

*ANCA, antineutrophil cytoplasmic antibody; CCI, Charlson comorbidity index*.

### Baseline Characteristics of AAV Subgroups

Comparison of patient characteristics of the AAV subgroups revealed no significant differences in terms of sex among patients with MPA, GPA and EGPA. However, among the AAV subgroups, patients with MPA had the oldest age when diagnosed, frequently diagnosed in ages 70–79 years. Meanwhile, GPA and EGPA were the most common in the age groups of 60–69 and 50–59, respectively. In addition, differences were noted in terms of income in the AAV subgroups, while the insurance types of patients were comparable. Patients with MPA had the highest mean CCI scores compared to patients with GPA and EGPA (Table 2).

**Table 2:** Comparison of characteristics between subgroup of patients with ANCA-associated vasculitis at diagnosis.

	**MPA**	**GPA**	**EGPA**	**ANCA-associated vasculitis**	***p*-value**
	**(*n* = 708)**	**(*n* = 638)**	**(*n* = 767)**	**(*n* = 2113)**	
**Sex**					
Male	305 (43.1)	300 (47.0)	370 (48.2)	975 (46.1)	0.121
Female	403 (56.9)	338 (53.0)	397 (51.8)	1138 (53.9)	
Age, years	64.3 ± 14.1	57.5 ± 15.2	52.9 ± 15.9	58.1 ± 15.8	<0.001
**Age distribution**					
10–19	11 (1.6)	9 (1.4)	14 (1.8)	34 (1.6)	<0.001
20–29	15 (2.1)	34 (5.3)	57 (7.4)	106 (5.0)	
30–39	22 (3.1)	47 (7.4)	87 (11.3)	156 (7.4)	
40–49	34 (4.8)	70 (11.0)	153 (19.9)	257 (12.2)	
50–59	112 (15.8)	152 (23.8)	178 (23.2)	442 (20.9)	
60–69	218 (30.8)	179 (28.1)	151 (19.7)	548 (25.9)	
70–79	246 (34.7)	126 (19.7)	104 (13.6)	476 (22.5)	
80–89	48 (6.8)	21 (3.3)	22 (2.9)	91 (4.3)	
≥90	2 (0.3)	0 (0.0)	1 (0.1)	3 (0.1)	
**Income^†^**					
<3 rd quintile	153 (21.9)	163 (26.0)	150 (19.8)	466 (22.4)	0.010
3~7 th quintile	212 (30.3)	213 (34.0)	264 (34.9)	689 (33.1)	
>7 th quintile	335 (47.9)	251 (40.0)	343 (45.3)	929 (44.6)	
**Insurance type**					
Employee	218 (30.8)	204 (32.0)	265 (34.6)	687 (32.5)	0.603
Self-employment	464 (65.5)	408 (63.9)	475 (61.9)	1347 (63.7)	
Medical-aid	26 (3.7)	26 (4.1)	27 (3.5)	79 (3.7)	
CCI	3.3 ± 1.6	2.8 ± 1.6	2.8 ± 1.4	3.0 ± 1.6	<0.001

† *Data are available for 700, 627, 757 and 2,084 patients with MPA, GPA, EGPA and ANCA-associated vasculitis, respectively*.

*Data are expressed as mean (SD) or frequencies (percentages)*.

*MPA, microscopic polyangiitis; GPA, granulomatosis with polyangiitis; EGPA, eosinophilic granulomatosis with polyangiitis; ANCA, antineutrophil cytoplasmic antibody; CCI, Charlson comorbidity index*.

### Annual Incidence, Prevalence, and Monthly and Seasonal Incidence of AAV

**Figure 1:**
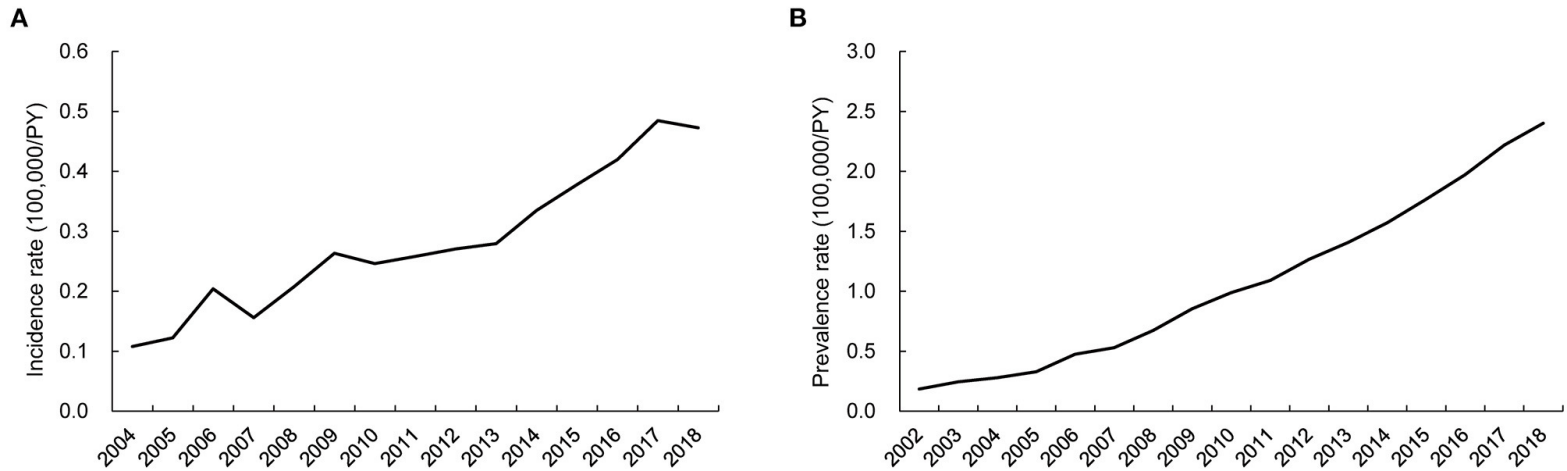
Time-trends of incidence and prevalence rate of ANCA-associated vasculitis. Annual trends of **(A)** incidence and **(B)** prevalence of AAV showing a continuous increase during the investigation period. ANCA, antineutrophil cytoplasmic antibody; PY, person-year.

**Figure 2:**
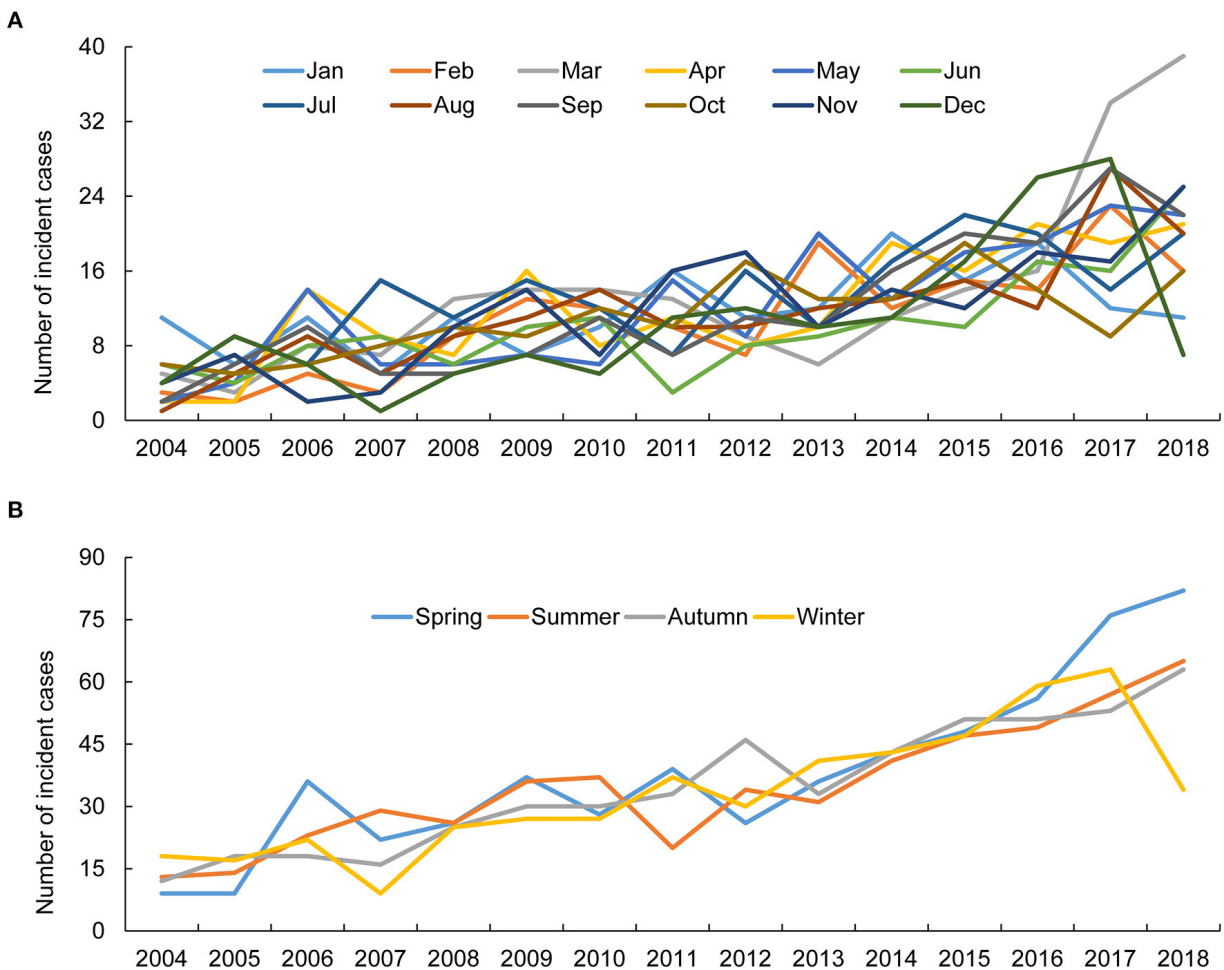
Monthly and seasonal incidence of ANCA-associated vasculitis. The difference in AAV incidence was not apparent according to the **(A)** month and **(B)** season. ANCA, antineutrophil cytoplasmic antibody.

The annual incidence and prevalence of AAV increased continuously during the observed years (both *p* < 0.001), and the incidence of MPA was the highest after 2015 among the AAV subgroups. In particular, the highest incidence of AAV was 0.48/100,000 person-years (PY) in 2017 and 2.40/100,000 PY in 2018 (Figures 1A,B). Incident cases of AAV were most frequently observed in March and lowest in June, but no significant monthly and seasonal differences were noted (*p* = 0.973 and *p* = 0.877 for every month and season, respectively; Figures 2A,B).

### Healthcare Cost and Outcomes in Patients with AAV

**Figure 3:**
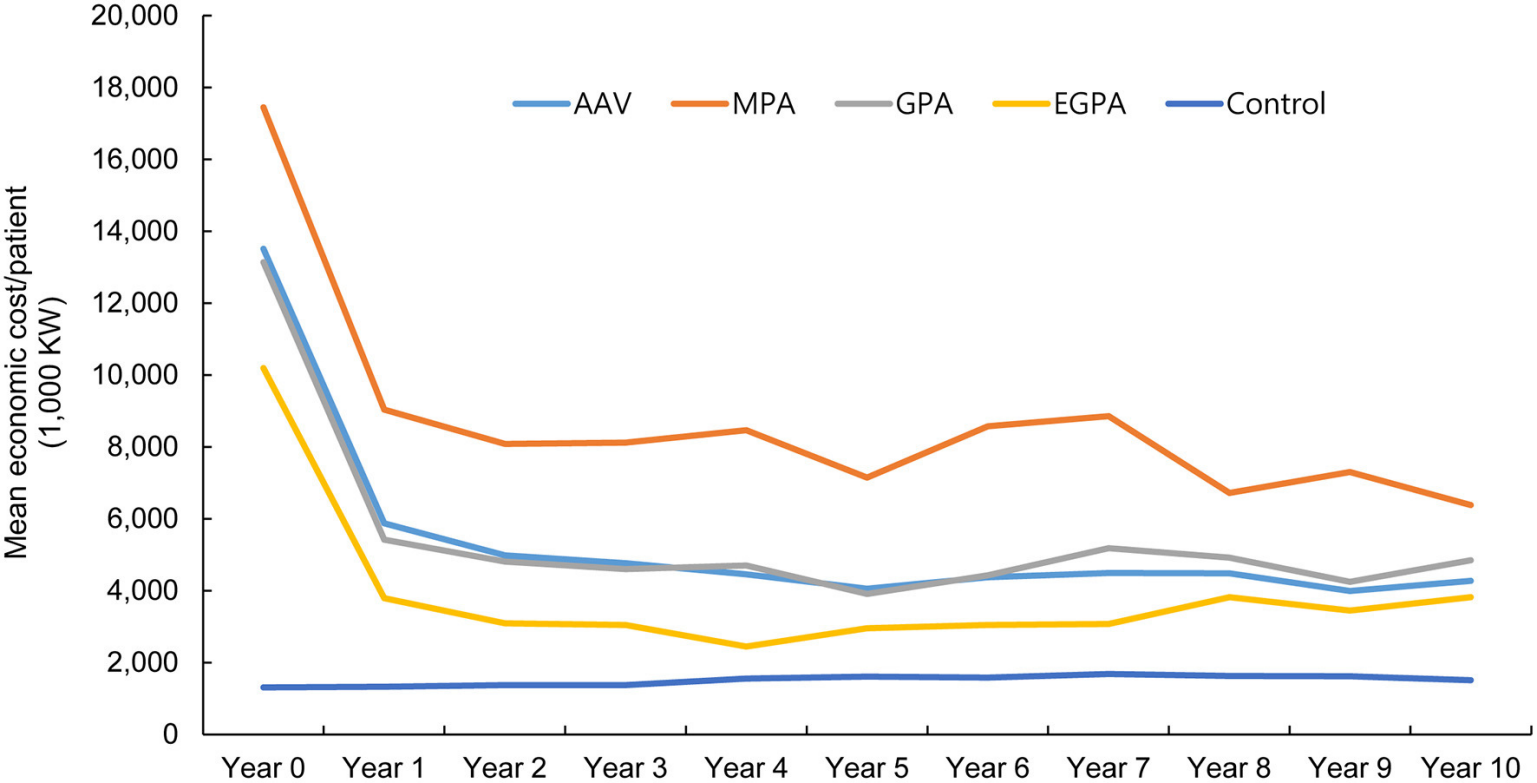
Estimation of annual economic cost in patients with ANCA-associated vasculitis and controls. While the economic cost in healthy controls remained stable during the follow-up period, patients with ANCA-associated vasculitis showed a steep reduction in medical costs in the second year after diagnosis, and remained similar after the third year. ANCA, antineutrophil cytoplasmic antibody; ESRD, end-stage renal disease; MPA, microscopic polyangiitis; GPA, granulomatosis with polyangiitis; EGPA, eosinophilic granulomatosis with polyangiitis.

Compared to the controls, the cost of medical care was greater in patients with AAV and highest in patients with MPA. This expenditure peaked in the first year of AAV diagnosis, rapidly declined in the second year, and was similar afterwards (Figure 3).

**Figure 4:**
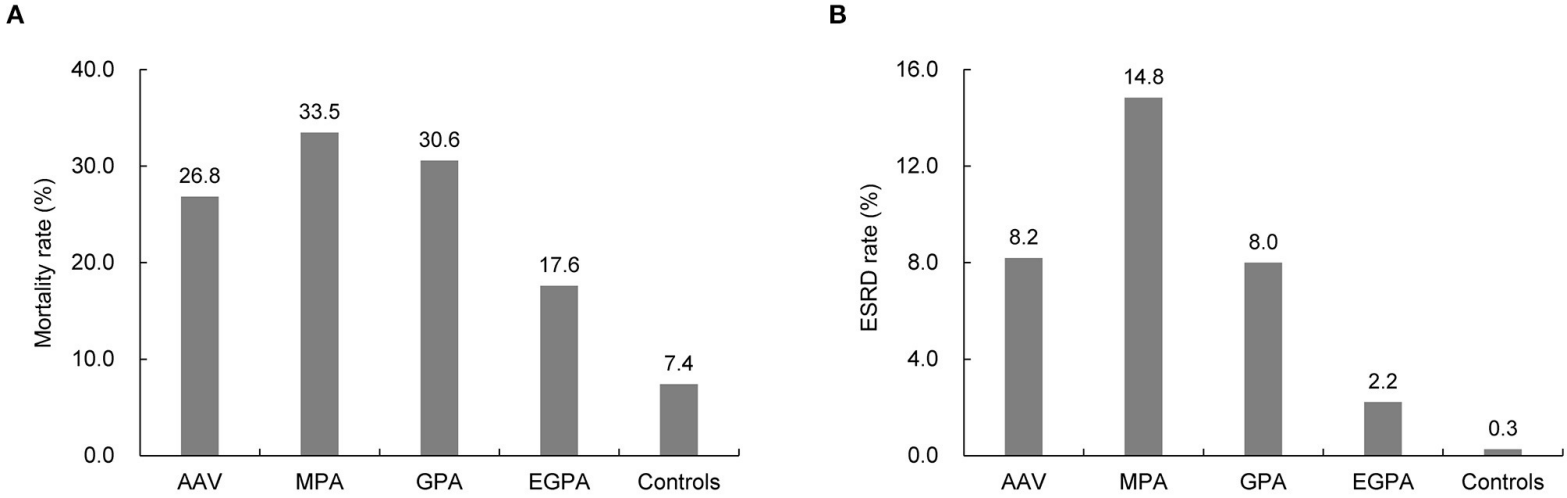
Comparison of mortality and ESRD rate between patients with ANCA-associated vasculitis and controls. Compared to the controls, patients with AAV had higher rates of **(A)** mortality and **(B)** developing ESRD. ESRD, end-stage renal disease; ANCA, antineutrophil cytoplasmic antibody; MPA, microscopic polyangiitis; GPA, granulomatosis with polyangiitis; EGPA, eosinophilic granulomatosis with polyangiitis.

Furthermore, during the mean follow-up period of 58.7 months, the reported overall mortality and ESRD rates of patients with AAV were 26.8 and 8.2%, respectively, which were significantly higher than those of the controls (all *p* < 0.001). The highest mortality and ESRD rates were found in patients with MPA (33.5 and 14.8%, respectively). The overall mortality and ESRD rates in patients with EGPA were the lowest in the AAV subgroups (17.6% and 2.2%, respectively) during the observation period (Figures 4A,B).

### Basal Characteristics of Patients with Mortality and ESRD

Regarding the clinical characteristics of patients with AAV related to mortality, patients who died tended to be male, older, and were mostly diagnosed with MPA. Furthermore, the proportion of those who were covered by medical aid care and baseline CCI was higher in patients who died (Table 3). In contrast, compared with patients who did not develop ESRD, those who developed ESRD were older, were mostly diagnosed with MPA, and had a higher CCI at disease diagnosis (Table 4).

**Table 3:** Baseline characteristics of ANCA-associated vasculitis patients suffering mortality and without.

	**Patients with mortality (*n* = 567)**	**Patients without mortality (*n* = 1546)**	***p*-value**
**Sex**			
Male	307 (54.1)	668 (43.2)	<0.001
Female	260 (45.9)	878 (56.8)	
Age, years	67.2 ± 11.9	54.7 ± 15.8	<0.001
**Diagnosis**			
MPA	237 (41.8)	471 (30.5)	<0.001
GPA	195 (34.4)	443 (28.7)	
EGPA	135 (23.8)	632 (40.9)	
**Income^†^**			
<3 rd quintile	132 (23.7)	334 (21.9)	0.228
3~7 th quintile	168 (30.2)	521 (34.1)	
>7 th quintile	257 (46.1)	672 (44.0)	
**Insurance type**			
Employee	194 (34.2)	493 (31.9)	<0.001
Self-employment	338 (59.6)	1009 (65.3)	
Medical-aid	35 (6.2)	44 (2.8)	
CCI	3.4 ± 1.6	2.8 ± 1.5	<0.001

† *Data were available for 557 and 1,527 patients with and without mortality, respectively*.

*Data are expressed as mean (SD) or frequencies (percentages)*.

*ANCA, antineutrophil cytoplasmic antibody; MPA, microscopic polyangiitis; GPA, granulomatosis with polyangiitis; EGPA, eosinophilic granulomatosis with polyangiitis; CCI, Charlson comorbidity index*.

**Table 4:** Basal characteristics of ANCA-associated vasculitis patients with and without ESRD.

	**Patients with ESRD (*n* = 173)**	**Patients without ESRD (*n* = 1940)**	***p*-value**
**Sex**			
Male	68 (39.3)	907 (46.8)	0.060
Female	105 (60.7)	1033 (53.2)	
Age, years	63.3 ± 14.9	57.6 ± 15.8	<0.001
**Diagnosis**			
MPA	105 (60.7)	603 (31.1)	<0.001
GPA	51 (29.5)	587 (30.3)	
EGPA	17 (9.8)	750 (38.7)	
**Income^†^**			
<3 rd quintile	37 (21.6)	429 (22.4)	0.633
3~7 th quintile	52 (30.4)	637 (33.3)	
>7 th quintile	82 (48.0)	847 (44.3)	
**Insurance type**			
Employee	53 (30.6)	634 (32.7)	0.525
Self-employment	111 (64.2)	1236 (63.7)	
Medical-aid	9 (5.2)	70 (3.6)	
CCI	3.5 ± 1.7	2.9 ± 1.5	<0.001

† *Data were available for 171 and 1,913 patients with and without ESRD, respectively*.

*Data are expressed as mean (SD) or frequencies (percentages)*.

*ANCA, antineutrophil cytoplasmic antibody; ESRD, end-stage renal disease; MPA, microscopic polyangiitis; GPA, granulomatosis with polyangiitis; EGPA, eosinophilic granulomatosis with polyangiitis; CCI, Charlson comorbidity index*.

## Discussion

Although accumulating evidence indicates that AAV is more common than in the past decades, the incidence and prevalence of AAV in Asia remain poorly understood. In the present study, the following results were obtained by searching through the NHID: (i) although AAV is still rare, the annual incidence and prevalence of AAV in South Korea showed a gradual rise, with the highest incidence and prevalence of 0.48/100,000 and 2.40/100,000 PY reported in 2017 and 2018, respectively; (ii) the economic healthcare burden of AAV was higher than that of the controls; this increased medical cost culminates in the first year of AAV diagnosis but remains stable after the third year of AAV diagnosis; and (iii) patients with AAV are at an increased risk of mortality and developing ESRD, which is significantly greater compared to the control group. To the best of our knowledge, this is the first study to evaluate the incidence, prevalence, and economic costs of AAV in Asia using a nationwide cohort.

The global incidence of AAV ranges 1.2–3.3/100,000 PY with a prevalence of 4.6–42.1/100,000 PY (4). While studies evaluating the incidence of AAV in Asia are rare, a single-district study from Japan demonstrated an average incidence of AAV of 22.6/million PY (8). Our results showed that the highest incidence and prevalence of AAV in South Korea were 4.8/million and 24.0/million PY, respectively, which is relatively lower than that reported in Japan. Meanwhile, a study performed in Taiwan using the National Health Insurance Database determined that the incidence of GPA was 0.37/million PY (13). The incidence of GPA in our study ranged 0.5–1/million PY, showing a similar numerical estimates with the study by Wu et al. In addition, in the present study, MPA was the most frequent diagnosis among the AAV cases after 2015, supporting that MPA is the most common disease subtype of AAV in Asia (14). Consistent with our data, an analysis of a national inpatient database from China demonstrated that MPA was the most common diagnosis requiring hospitalization, although the incidence and prevalence of AAV could not be calculated in the study by Li et al. (15). However, even in regions within Asia, an inconsistency of patient clinical characteristics has been also reported. For example, single-center studies from India reported a relatively lower age of onset in patients with GPA with a high positivity rate for anti PR3 (or c-ANCA), and a distinctive clinical feature was observed compared to the Western cohorts (16, 17). Altogether, it is apparent that there still is an uncertainty regarding the characteristics of patients with AAV in Asia (18), highlighting the needs for a further exploration.

A previous investigation indicated that ultraviolet radiation has an inverse correlation with the incidence of AAV, suggesting that seasonal variation could be related to disease incidence (19). Nonetheless, in our study, while the number of AAV cases was highest in March, the influence of season on the incidence of AAV was not significant, which was found similar in a recent national Scottish registry-based study, confirming a non-significant association between seasonality and AAV incidence (20). On our study, the overall incidence and prevalence of AAV seemed to increase gradually during the observation period. These results might have been attributed to increased disease awareness and patient management strategies among physicians attending to patient care. However, the incidence and prevalence of AAV appeared to be lower than those reported in Western countries, implying that a geographic distinction exists.

Patients with autoimmune diseases, such as rheumatoid arthritis and systemic lupus erythematosus, are subject to increased healthcare utilization and economic costs (21, 22). In line with this finding, a retrospective study from Italy revealed that healthcare costs in patients with AAV were noticeably high (23). On analyzing the trends of average medical costs of patients with AAV, we found that their economic expenditure was remarkably high in the first year after disease diagnosis and decreased sharply in the second year. Meanwhile, medical costs appeared to be similar after the third year of AAV diagnosis. Among the AAV subtypes, patients with MPA had the highest medical expenditure, which might be due to the fact that patients with MPA are more likely to undergo intensive medical care owing to the greater risk of death and development of ESRD, as described previously and in the present study (24, 25). Conversely, patients with EGPA had the lowest medical expenditure among AAV subtypes, which could be explained by the favorable prognoses of patients with EGPA (26). The medical cost of the control group remained constant in 10 years and was lower than that of patients with AAV, irrespective of disease subtypes. Finally, our study revealed a higher frequency of death in patients with AAV among medical-aid covered individuals. Taken together, our findings emphasize that programs that reduce the medical expenses of patients with AAV should be continuously implemented to facilitate adequate medical care.

Although significant advances have been made in the diagnosis and treatment of AAV, there is a heightened risk of mortality and ESRD in patients with AAV. The European Vasculitis Society (EUVAS) data showed that the 1- and 5-year survival rates in patients with AAV were 88 and 78%, respectively (27). Furthermore, long-term data from the EUVAS clinical trial registry reported that 18.6% and 13% of patients died and developed ESRD, respectively (28). Additionally, the analyses of the Glomerular Disease Collaborative Network inception cohort described a 5-year mortality and ESRD rates of 28 and 46%, respectively, in patients with AAV, although a substantial improvement in patient prognoses was also observed compared to previous years (29). In our study population, 26.8% of the patients died and 8.2% developed ESRD, which was significantly higher than that in the control group. Collectively, these findings indicate that AAV is a life-threatening disease that requires prompt diagnosis and timely treatment.

This study has some limitations. First, given that the results of laboratory tests were not recorded in the NHID, we were not able to investigate the proportion of ANCA-positive patients and the differences in ANCA specificity and laboratory features according to the AAV subtypes. Second, to increase diagnostic accuracy, we only included patients with AAV who had the corresponding ICD-10 code and V code for rare and intractable diseases or those who were treated with glucocorticoids, which might have led to disease underestimation. Third, due to the limitations of the NHID, disease-specific details of patients with AAV, such as the extent of disease (e.g. limited or systemic), organ involvement patterns, and the existence of organ damage, were not available. Fourth, the information and influence of medications, besides glucocorticoids, to treat AAV could not be evaluated. Fifth, the description of cause-specific death in our patients could not be provided, owing to the inherent limitation of the NHID. We believe that additional research are necessary in the future to better understand the epidemiology and characteristics of patients with AAV, especially in Asia.

In conclusion, the present study identified a secular trend of increasing incidence and prevalence in South Korea. It was also demonstrated that patients with AAV required greater expenditure in terms of medical care and conferred a higher risk of death and development of ESRD compared to the controls. Our findings provide valuable information for understanding the epidemiology of AAV in Asia and highlight the importance of early recognition and implementation of optimal treatment for AAV to improve patient outcomes.

## Data Availability Statement

The datasets presented in this article are not readily available. Data are available from the Korea National Health Insurance Sharing Service (NHISS) for licensed researchers. However, data are restricted to be shared publicly owing to the sensitive nature of the collected data. Requests to access the datasets should be directed to contact at: https://nhiss.nhis.or.kr, contact: +82-33-736-2432, 2433.

## Ethics Statement

The studies involving human participants were reviewed and approved by Institutional Review Board of the NHIS Ilsan Hospital. Written informed consent for participation was not required for this study in accordance with the national legislation and the institutional requirements.

## Author Contributions

Conceptualization: SA and J-SP. Methodology, validation, investigation, and project administration: SA, HL, and J-SP. Software and visualization: SA and HL. Formal analysis and data curation: HL. Resources: CL and Y-BP. Writing—original draft preparation: SA, CL, Y-BP, and J-SP. Writing—review and editing: SA, HL, CL, Y-BP, J-SP, and S-WL. Supervision: CL, Y-BP, and S-WL. Funding acquisition: S-WL. All authors have read and agreed to the final version of the manuscript.

## Funding

This work was supported by a faculty research grant of Yonsei University College of Medicine (6-2019-0184), a grant from the Korea Health Technology R&D Project through the Korea Health Industry Development Institute, funded by the Ministry of Health and Welfare (HI14C1324), and the Handok Inc., Seoul, Republic of Korea (HANDOK 2021-006).

## Conflict of Interest

The authors declare that the research was conducted in the absence of any commercial or financial relationships that could be construed as a potential conflict of interest.

## Publisher's Note

All claims expressed in this article are solely those of the authors and do not necessarily represent those of their affiliated organizations, or those of the publisher, the editors and the reviewers. Any product that may be evaluated in this article, or claim that may be made by its manufacturer, is not guaranteed or endorsed by the publisher.
